# Supplementary material for: Infrapatellar Fat Pad Modulates Osteoarthritis-Associated Cytokine and MMP Expression in Human Articular Chondrocytes
Source: Cells. 2023 Dec 15;12(24):2850. doi: 10.3390/cells12242850 (PMC10741519; doi:10.3390/cells12242850)
Supplement: Supplementary file 1 [file cells-12-02850-s001.zip › cells-2746877-supplementary.pdf]

Array C6 single values n=4 per group

	FP CO1	FP CO2	FP CO3	FP CO4	FP OA1	FP OA2	FP OA3	FP OA4
Angiogenin	1.097	0.869	1.149	1.139	1.923	1.137	1.247	1.372
Leptin	0.150	0.675	0.196	0.518	0.209	1.274	0.632	0.562
RANTES (CCL5)	0.333	0.279	0.209	0.497	0.061	1.480	0.681	0.351
FGF-6	0.388	0.247	0.488	0.606	0.566	0.542	0.089	0.535
LIGHT (TNFSF14)	0.144	0.467	0	0.458	0.280	1.088	0.484	0.390
MCP-1 (CCL2)	0.372	0.258	0	0.439	0.368	0.890	0.424	0.282
SDF1a (CXCL12)	0	0.122	0.086	0.518	0.219	1.162	0.554	0.167
SCF	0	0.074	0.074	0.463	0.134	1.192	0.574	0.172
PDGF-BB	0.179	0.155	0	0.344	0	1.094	0.458	0.240
Eotaxin-2 (CCL24)	0.301	0.323	0.434	0.322	0.199	0.318	0	0.402
TARC (CCL17)	0	0.045	0.035	0.487	0.218	0.890	0.475	0.137
NAP-2 (CXCL7)	0.757	0	0	0.124	0.383	0.226	0	0.190
IGF-1	0	0.492	0	0.274	0.085	0.391	0	0.252
Eotaxin-1 (CCL11)	0.175	0	0	0.586	0.260	0	0	0.343
NT-3	0.058	0	0	0.293	0.465	0.318	0	0.220
MCP-2 (CCL8)	0.034	0.049	0	0.270	0.266	0.458	0.210	0
BDNF	0	0	0.291	0.279	0.194	0.185	0.081	0.230
Eotxin-2 (CCL26)	0.230	0	0.141	0.255	0.181	0.200	0	0.228
IL-1 ra (IL-1 F3)	0	0	0	0.331	0.529	0.326	0	0
CNTF	0	0	0.253	0.337	0.125	0.088	0	0.379
TGF beta 3	0	0	0	0.241	0.142	0.377	0.248	0.037
TNF alpha	0	0	0	0.255	0.185	0.299	0.210	0.052
PARC (CCL18)	0	0	0	0.341	0.427	0.222	0	0
TNF beta	0	0	0	0.257	0.240	0.243	0.143	0.077
IL-15	0	0	0	0.358	0.362	0.179	0	0
EGF	0	0	0.053	0.365	0.168	0	0	0.274
TGF beta 1	0	0	0	0.164	0.049	0.397	0.212	0
M-CSF	0.048	0	0	0.259	0.315	0.173	0	0
Ck beta 8-1 (CCL23)	0	0	0.198	0.191	0.043	0.047	0	0.294
IGFBP-4	0	0	0	0.290	0.287	0	0	0.152
MCP-3 (MARC/CCL7)	0	0	0	0.172	0.203	0.224	0.085	0
BMP-4	0	0	0.149	0.171	0.057	0.055	0	0.224
IL-1 beta (IL-1F2)	0	0	0	0.198	0.325	0.132	0	0
MCP-4 (CCL13)	0	0	0	0.208	0.234	0.156	0.039	0
IGFBP-2	0	0	0	0.180	0.227	0	0	0.213
FGF-7 (KGF)	0	0	0	0.284	0.201	0.128	0	0
Fractalkine (CX3CL1)	0	0	0	0.264	0.236	0.092	0	0
GDNF	0	0	0	0.191	0.259	0.079	0	0
Flt-3 Ligand	0	0	0	0.241	0.189	0.084	0	0
IFN-gamma	0	0	0	0.090	0.223	0.070	0	0.101
IL-16	0	0	0	0.168	0.255	0.045	0	0
MDC (CCL22)	0	0	0	0.129	0.230	0.102	0	0
BLC (CXCL13)	0	0	0	0.203	0.073	0.065	0	0.113
GCP-2 (CXCL6)	0	0	0	0.186	0.190	0.057	0	0
IL-1 alpha (IL-1F1)	0	0	0	0.120	0.222	0.054	0	0
IL-13	0	0	0	0.115	0.102	0.085	0	0
IL-10	0	0	0	0.087	0.059	0.139	0	0
IL-3	0	0	0	0	0.210	0.053	0	0
MIG (CXCL9)	0	0	0	0	0.149	0.090	0	0
MIP3 alpha (CCL20)	0	0	0	0	0.155	0.077	0	0
IL-2	0	0	0	0	0.176	0.055	0	0
IL-4	0	0	0	0	0.155	0.035	0	0
IGFBP-1	0	0	0	0.041	0.103	0	0	0.040
MIP-1 delta (CCL15)	0	0	0	0	0.118	0.066	0	0
IL-7	0	0	0	0.103	0.078	0	0	0
BMP-6	0	0	0	0.037	0	0	0	0.130
IL-5	0	0	0	0	0.113	0	0	0
I-309 (CCL1)	0	0	0	0	0.094	0	0	0
GM-CSF	0	0	0	0	0.089	0	0	0
IL-6	0	0	0	0	0.088	0	0	0

increasing optical density

0.2 0.4 0.6 0.8 1.0 1.2 1.4 1.6 1.8

mean normalized chemiluminescence signal

	FP CO1	FP CO2	FP CO3	FP CO4	FP OA1	FP OA2	FP OA3	FP OA4
Adiponectin (ACRP 30)		1.741	3.164	1.891	2.999	3.331	2.882	2.679
TIMP-2		0.711	3.364	1.596	2.085	1.783	2.065	2.382
MIF		0.038	1.576	0.802	0.671	0.563	0	1.135
TIMP-1		0	1.932	0.316	0.616	0.720	0.074	1.010
ICAM-1 (CD54)		0.599	1.595	0.634	0.329	0.504	0.038	0.739
OSM		0.060	1.227	0.512	0.999	0.907	0	0.381
TECK (CCL25)		0	1.782	0.291	0.202	1.086	0	0.518
EGFR		0.470	1.001	0.776	0.470	0.453	0	0.680
TRAIL R3 (TNFRSF10C)		0	0.915	0.397	0.770	0.751	0	0.496
IGFBP-6		0	0.346	0.325	0.640	0.072	0	1.584
MIP-1 alpha (CCL3)		0	1.066	0.445	0.512	0.399	0	0.490
TNF RI (TNFRSF1A)		0.265	0.762	0.134	0.882	0.552	0	0.235
gp130		0	0.827	0.224	0.787	0.702	0	0.135
I-TAC (CXCL11)		0.291	0.467	0.618	0.123	0.785	0	0.272
TPO		0	0.998	0.303	0.548	0.393	0	0.289
MIP-1 beta (CCL14)		0	0.887	0.358	0.602	0.274	0	0.274
TNF RII (TNFRSF1B)		0	0.903	0.140	0.678	0.555	0	0.081
TRAIL R4 (TNFRSF10D)		0	0.635	0.280	0.641	0.445	0	0.193
uPAR		0	0.792	0.251	0.627	0.310	0	0.197
CTACK (CCL27)		0.582	0.323	0.531	0	0.217	0	0.350
IL-6 R		0	0.481	0.153	0.641	0.719	0	0
PLGF		0	0.762	0.179	0.588	0.381	0	0
IGFBP-3		0.116	0.481	0.363	0.261	0.224	0	0.364
HCC-4 (CCL16)		0	0.097	0.090	0.602	0.899	0	0
VEGF-A		0	0.626	0.229	0.544	0.237	0	0
Lymphotaktin		0	1.090	0.302	0.172	0	0	0.031
MSP alpha/beta		0	0.739	0.281	0.512	0.044	0	0
NT-4		0	0.785	0.286	0.478	0	0	0
Dtk		0.388	0.479	0.320	0.116	0	0	0.235
VEGF-D		0	0.521	0.237	0.448	0.322	0	0
MIP3 beta (CCL19)		0	0.702	0.234	0.476	0	0	0
OPG (TNFRSF11B)		0	0.797	0.197	0.412	0	0	0
CCL28 (MEC)		0	0	0.099	0.235	1.033	0	0
IL-2 R alpha		0	0.471	0.049	0.419	0.356	0	0
FGF-9		0	0.125	0.253	0.480	0.346	0	0.061
Fas (Apo-1)		0	0.303	0.321	0.266	0.217	0	0
IGF-1R		0	0.371	0.238	0.287	0	0	0.053
ENA-78 (CXCL5)		0.042	0.250	0.291	0.197	0.097	0	0
HGF		0	0	0	0.280	0.582	0	0
IL-1 R4 (ST2)		0	0.341	0.205	0.290	0	0	0
AR		0	0	0.075	0.286	0.470	0	0
IL-17A		0	0.415	0	0.340	0.057	0	0
ANGPT2		0	0.246	0.053	0.329	0.179	0	0
GRO alpha (CXCL1)		0	0.096	0	0.408	0.270	0	0
Axl		0	0	0.113	0.293	0.358	0	0
FGF-4		0	0.124	0.226	0.280	0.130	0	0
BTC		0	0	0	0.179	0.575	0	0
IL-12 p70		0	0.431	0	0.323	0	0	0
IL-12 p40		0	0.358	0.049	0.276	0	0	0
ICAM-3 (CD50)		0.125	0.403	0.131	0	0	0	0
IL-11		0	0.328	0.076	0.245	0	0	0
IL-1 R1		0	0.285	0.126	0.232	0	0	0
bFGF		0	0	0.078	0.280	0.196	0	0
GRO a/b/c		0	0.099	0	0.343	0.061	0	0
AgRP		0	0	0.083	0.218	0.145	0.034	0
GITR Ligand (TNFSF18)		0	0.069	0.061	0.346	0	0	0
IL-8 (CXCL8)		0	0	0	0.297	0.146	0	0
GITR (TNFRSF18)		0	0.059	0	0.352	0	0	0
G-CSF		0	0	0	0.263	0	0	0
beta-NGF		0	0	0	0.185	0	0	0

increasing optical density

0.2 0.4 0.6 0.8 1.0 1.2 1.4 1.6 1.8 2.0 2.2 2.4 2.6 2.8 3.0 3.2

mean normalized chemiluminescence signal

Array C7 single values n=3/4 per group

CO1-Donor-Array was not usable for analysis

Array C6 mean values n=4 per group

	Mean FP CO	Mean FP OA
Angiogenin	1.064	1.420
FGF-6	0.432	0.433
Leptin	0.385	0.669
Eotaxin-2 (CCL24)	0.345	0.230
RANTES (CCL5)	0.330	0.643
MCP-1 (CCL2)	0.267	0.491
LIGHT (TNFSF14)	0.267	0.561
NAP-2 (CXCL7)	0.220	0.200
IGF-1	0.192	0.182
Eotaxin-1 (CCL11)	0.190	0.151
SDF1a (CXCL12)	0.181	0.525
PDGF-BB	0.170	0.448
Eotxin-2 (CCL26)	0.156	0.152
SCF	0.153	0.518
CNTF	0.148	0.148
BDNF	0.142	0.172
TARC (CCL17)	0.142	0.430
EGF	0.105	0.110
Ck beta 8-1 (CCL23)	0.097	0.096
IL-15	0.090	0.135
MCP-2 (CCL8)	0.088	0.233
NT-3	0.088	0.251
PARC (CCL18)	0.085	0.162
IL-1 ra (IL-1 F3)	0.083	0.214
BMP-4	0.080	0.084
M-CSF	0.077	0.122
IGFBP-4	0.072	0.110
FGF-7 (KGF)	0.071	0.082
Fractalkine (CX3CL1)	0.066	0.082
TNF beta	0.064	0.176
TNF alpha	0.064	0.187
TGF beta 3	0.060	0.201
Flt-3 Ligand	0.060	0.068
MCP-4 (CCL13)	0.052	0.107
BLC (CXCL13)	0.051	0.063
IL-1 beta (IL-1F2)	0.050	0.114
GDNF	0.048	0.084
GCP-2 (CXCL6)	0.046	0.062
IGFBP-2	0.045	0.110
MCP-3 (MARC/CCL7)	0.043	0.128
IL-16	0.042	0.075
TGF beta 1	0.041	0.165
MDC (CCL22)	0.032	0.083
IL-1 alpha (IL-1F1)	0.030	0.069
IL-13	0.029	0.047
IL-7	0.026	0.019
IFN-gamma	0.022	0.099
IL-10	0.022	0.050
IGFBP-1	0.010	0.036
BMP-6	0.009	0.032
IL-3	0	0.066
MIG (CXCL9)	0	0.060
MIP3 alpha (CCL20)	0	0.058
IL-2	0	0.058
IL-4	0	0.048
MIP-1 delta (CCL15)	0	0.046
IL-5	0	0.028
I-309 (CCL1)	0	0.024
GM-CSF	0	0.022
IL-6	0	0.022

increasing optical density

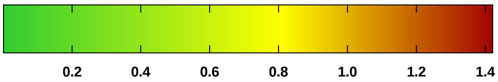

mean normalized chemiluminescence signal

	Mean FP CO	Mean FP OA
Adiponectin (ACRP 30)	2.265	2.973
TIMP-2	1.890	2.079
ICAM-1 (CD54)	0.943	0.402
MIF	0.805	0.592
TIMP-1	0.749	0.605
EGFR	0.749	0.401
TECK (CCL25)	0.691	0.452
OSM	0.599	0.572
MIP-1 alpha (CCL3)	0.503	0.350
CTACK (CCL27)	0.479	0.142
Lymphotaktin	0.464	0.051
I-TAC (CXCL11)	0.459	0.295
TRAIL R3 (TNFRSF10C)	0.437	0.504
TPO	0.434	0.307
MIP-1 beta (CCL14)	0.415	0.288
Dtk	0.395	0.088
TNF RI (TNFRSF1A)	0.387	0.417
NT-4	0.357	0.120
gp130	0.350	0.406
TNF RII (TNFRSF1B)	0.348	0.328
uPAR	0.348	0.283
MSP alpha/beta	0.340	0.139
OPG (TNFRSF11B)	0.331	0.103
IGFBP-3	0.320	0.212
PLGF	0.314	0.242
MIP3 beta (CCL19)	0.312	0.119
TRAIL R4 (TNFRSF10D)	0.305	0.320
VEGF-A	0.285	0.195
VEGF-D	0.252	0.192
IGFBP-6	0.224	0.574
ICAM-3 (CD50)	0.220	0
IL-6 R	0.212	0.340
Fas (Apo-1)	0.208	0.121
IGF-1R	0.203	0.085
ENA-78 (CXCL5)	0.194	0.074
IL-1 R4 (ST2)	0.182	0.072
IL-2 R alpha	0.173	0.194
IL-12 p70	0.144	0.081
IL-17A	0.138	0.099
IL-1 R1	0.137	0.058
IL-12 p40	0.136	0.069
IL-11	0.135	0.061
FGF-9	0.126	0.222
FGF-4	0.117	0.103
ANGPT2	0.100	0.127
HCC-4 (CCL16)	0.062	0.375
GITR Ligand (TNFSF18)	0.043	0.086
Axl	0.038	0.163
GRO a/b/c	0.033	0.101
CCL28 (MEC)	0.033	0.317
GRO alpha (CXCL1)	0.032	0.170
AgRP	0.028	0.099
bFGF	0.026	0.119
AR	0.025	0.189
GITR (TNFRSF18)	0.020	0.088
HGF	0	0.215
BTC	0	0.189
IL-8 (CXCL8)	0	0.111
G-CSF	0	0.066
beta-NGF	0	0.046

Array C7 mean values n=3/4 per group

CO1-Donor-Array was not usable for analysis

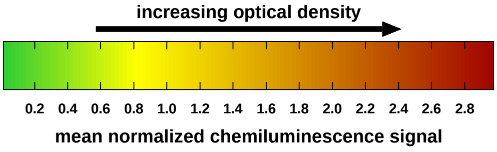

Figure S1. Heatmaps for Raybiotech Human Cytokine Array C1000 (consists of C6 and C7 Array and detects 120 Human Cytokines 2x60).
